# Maternal predator‐exposure affects offspring size at birth but not telomere length in a live‐bearing fish

**DOI:** 10.1002/ece3.6035

**Published:** 2020-01-28

**Authors:** Stefano Monteforte, Silvia Cattelan, Chiara Morosinotto, Andrea Pilastro, Alessandro Grapputo

**Affiliations:** ^1^ Department of Biology University of Padova Padova Italy; ^2^ Bioeconomy Research Team Novia University of Applied Sciences Ekenäs Finland

**Keywords:** environmental stress, maternal effects, *Poecilia reticulata*, predation risk, telomere

## Abstract

The perception of predation risk could affect prey phenotype both within and between generations (via parental effects). The response to predation risk could involve modifications in physiology, morphology, and behavior and can ultimately affect long‐term fitness. Among the possible modifications mediated by the exposure to predation risk, telomere length could be a proxy for investigating the response to predation risk both within and between generations, as telomeres can be significantly affected by environmental stress. Maternal exposure to the perception of predation risk can affect a variety of offspring traits but the effect on offspring telomere length has never been experimentally tested. Using a live‐bearing fish, the guppy (*Poecilia reticulata*), we tested if the perceived risk of predation could affect the telomere length of adult females directly and that of their offspring with a balanced experimental setup that allowed us to control for both maternal and paternal contribution. We exposed female guppies to the perception of predation risk during gestation using a combination of both visual and chemical cues and we then measured female telomere length after the exposure period. Maternal effects mediated by the exposure to predation risk were measured on offspring telomere length and body size at birth. Contrary to our predictions, we did not find a significant effect of predation‐exposure neither on female nor on offspring telomere length, but females exposed to predation risk produced smaller offspring at birth. We discuss the possible explanations for our findings and advocate for further research on telomere dynamics in ectotherms.

## Graphical abstract

Telomere length could be a proxy for investigating the response to predation risk both within and between generations. We tested if the perception of predation risk could affect the telomere length of adult females and their offspring using the guppy, *Poecilia reticulata*, a live‐bearing fish. Contrary to our predictions, we did not find a significant effect of predation‐exposure neither on female mothers nor on offspring telomere length, but female mothers exposed to predation risk produced smaller offspring at birth.

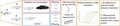

## 1 INTRODUCTION

Predator‐induced phenotypic plasticity is ubiquitous in nature and is known to trigger a range of flexible responses that aim to minimize the risk of being preyed upon. Such responses vary from defensive morphologies to behavioral compensation, to changes in life‐history traits and strategies (Hawlena & Schmitz, 2010; Khater, Murariu, & Gras, 2016; Relyea, 2001). One of many examples is found in the crucian carp (*Carassius carassius*), which develops an increased deeper body morphology when exposed to piscivorous fishes, an inducible morphological defense against gape‐limited predators (Brönmark & Miner, 1992). An induced response can be found also in the mosquito *Culex pipiens*, in which individuals that developed under predation risk are larger in size and disperse further (Alcalay, Tsurim, & Ovadia, 2018). Predator‐induced phenotypic changes are not limited to the individuals that directly experience the predation risk, but they can also arise in the offspring via maternal effects (Mousseau & Fox, 1998; Räsänen & Kruuk, 2007).

The transmission of information via maternal effects can be adaptive for the offspring if the acquired phenotype increases their chance of survival, due to morphological (e.g. Agrawal, Laforsch, & Tollrian, 1999; Tollrian, 1995) or behavioral (e.g. Storm & Lima, 2010; Zhang, Parent, Weaver, & Meaney, 2004) modifications. Mothers can influence offspring phenotypes through differential allocation of resources in terms of nutrients, hormones, and antibodies to their eggs or embryos, enhancing the ability of the offspring to cope with the adverse environment they will face (Bestion, Teyssier, Aubret, Clobert, & Cote, 2014; Giesing, Suski, Warner, & Bell, 2011; Morosinotto et al., 2013). For example, in the three‐spined stickleback (*Gasterosteus aculeatus*), the presence of predators prior to egg deposition resulted in offspring with an increased antipredator behavior (Giesing et al., 2011). Maternal effects, however, do not always result in a benefit for the progeny: maternal stress could lead to an increased concentration of glucocorticoid hormones in the eggs that can negatively influence offspring phenotype and viability (McGhee, Pintor, Suhr, & Bell, 2012; Saino, Romano, Ferrari, Martinelli, & Møller, 2005).

The perception of predator presence or of prey alarm cues in the nearby environment constitutes a great source of stress for the potential prey. Physiological stress is generally an adaptive syndrome that consists of a set of behavioral and physiological adjustments geared to increase survivorship during life‐threatening situations and to maintain physiological homeostasis, although at some cost (Wingfield & Ramenofsky, 1999). The stress response generally results in the secretion of glucocorticoids (Wingfield & Sapolsky, 2003), of which cortisol is the main product in teleost fish (Schreck, Bradford, Fitzpatrick, & Patiño, 1989), and their increase of the plasmatic levels (Campeau, Nyhuis, Sasse, Day, & Masini, 2008; Cockrem & Silverin, 2002), which, in turn, will increase the oxidative stress level (Haussmann, Longenecker, Marchetto, Juliano, & Bowden, 2012; Lin, Decuypere, & Buyse, 2004). A prolonged period of heightened oxidative stress is known to impair cellular and organismal functions (Guachalla & Rudolph, 2010) and negatively impact fitness of individuals in the long‐term (Slos & Stoks, 2008). For instance, previous studies have shown that early presence of glucocorticoids in the eggs negatively affects offspring size at birth (Eriksen, Bakken, Espmark, Braastad, & Salte, 2006; McCormick, 1998), with potential detrimental effects for survival (Dial, Reznick, & Brainerd, 2016).

Among the possible negative effects that the stress can have on fitness, telomere length seems to assume an important role. Telomeres are nucleoprotein complexes that cap the ends of linear chromosomes of eukaryotes (Blackburn, 2001). Telomeric nucleotide repeats shorten at each cell cycle until telomeres reach a critical size and ultimately limit cell growth (Shay & Wright, 2007). This entails for the observed telomere shortening with age in both mammals and birds (Haussmann et al., 2003), while studies on ectotherms have yielded mixed results (Hartmann et al., 2009; Hatakeyama et al., 2008; Lund, Glass, Tolar, & Blazar, 2009; reviewed in Olsson, Wapstra, & Friesen, 2018). Telomeres are particularly susceptible to oxidative damages because of their high guanine content (Monaghan, 2010); thus, oxidative stress is thought to be the most relevant cause of telomere attrition in wild vertebrates (Haussmann & Marchetto, 2010; von Zglinicki, 2002). Stress exposure might increase the rate of telomere loss via oxidative damages (e.g. Barnes, Fouquerel, & Opresko, 2019; Chatelain, Drobniak, & Szulkin, 2020; Epel et al., 2004; Kotrschal, Ilmonen, & Penn, 2007; Reichert & Stier, 2017), which in turn may increase aging rate and hence shortening life span (Heidinger et al., 2012; Wilbourn et al., 2018). Despite the increasing interest in studying telomere dynamics (Monaghan, Eisenberg, Harrington, & Nussey, 2018), the effect of environmental stress on telomere attrition in ectotherms is still largely unknown (Angelier, Costantini, Blévin, & Chastel, 2018; Chatelain et al., 2020; Olsson et al., 2018). One reason why understanding telomere dynamics is often difficult is that maternal effects are known to affect offspring phenotype including offspring telomere length (Asghar, Bensch, Tarka, Hansson, & Hasselquist, 2015; Haussmann & Heidinger, 2015; Marasco, Boner, Griffiths, Heidinger, & Monaghan, 2019) which, along with environmental stressors, determines the subsequent telomere dynamic in the adulthood (Heidinger et al., 2012).

**Figure 1:**
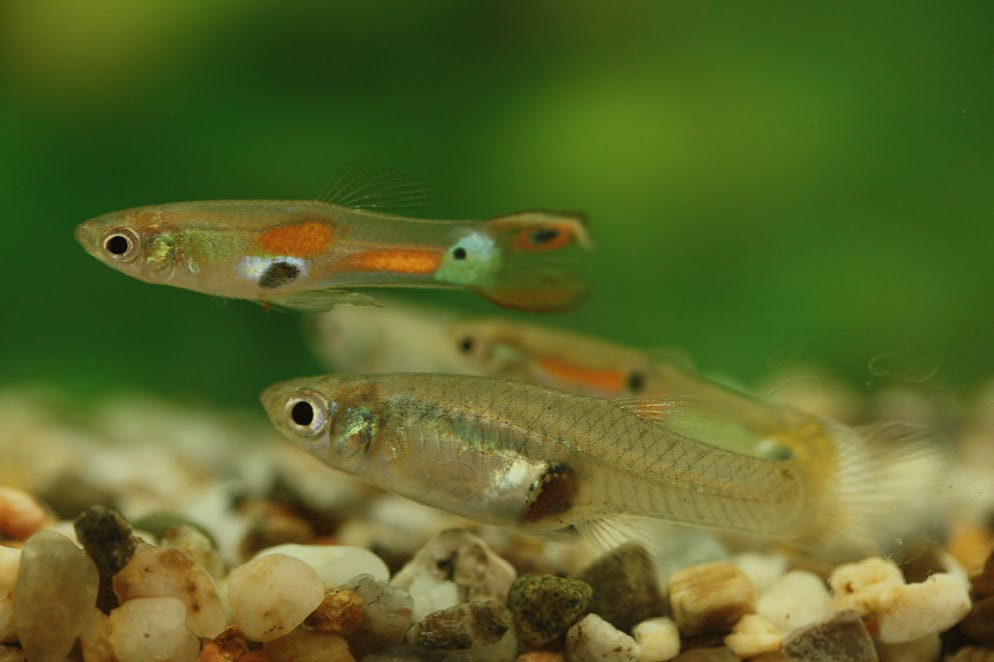
A bright‐colored male and a female guppy, *Poecilia reticulata*, descendants from a high predation site of the Tacarigua river (Trinidad). Credits: Clelia Gasparini

In our study, we investigated the effect of the perception of predation risk on the telomere length of female guppies, *Poecilia reticulata*, and the potential effects on their offspring in terms of offspring telomere length and body size. Guppies are live‐bearing fish (Figure 1) that have been extensively used as a model organism in ecological, behavioral, and evolutionary studies (Magurran, 2005). Predation plays a central role in shaping a range of antipredator traits in wild guppy population, including evolutionary changes in life‐history traits (Endler, 1995; Magurran, 2005; Reznick, Bryga, & Endler, 1990). Predation risk may also induce plastic behavioral modifications, as shown by the increased schooling tendency (a common fish antipredator behavior) of guppies transplanted from low to high predation regime environments (Magurran, Seghers, Carvalho, & Shaw, 1992) or by the presence of a typical inspection to the predator (Dugatkin, 1988). The guppy is also suitable for testing maternal effects on offspring because mothers may influence offspring phenotype during gestation (Reznick, Callahan, & Llauredo, 1996), by affecting, for example, their risk‐taking behaviors (White & Wilson, 2019).

We exposed females to visual predator cue and conspecific alarm during the development of the embryos controlling for both maternal and paternal contribution. We then measured female telomere length, offspring telomere length, and body size soon after birth. There is evidence that the predator presence or predation attempts can have an effect on telomere length of the exposed individuals (Burraco, Díaz‐Paniagua, & Gomez‐Mestre, 2017; Kärkkäinen et al., 2019; McLennan et al., 2016; Olsson, Pauliny, Wapstra, & Blomqvist, 2010), thus suggesting that both females and offspring telomere length will be affected. However, no study has experimentally investigated the effect of the perception of predation risk on both female and offspring telomere length. We thus predict that (a) females exposed to predation risk would experience a higher stress due to the perceived predation risk and will consequently exhibit shorter telomere length. Previous studies showed that maternal stress can negatively affect egg size and/or offspring size at birth (Coslovsky & Richner, 2011; Donelan & Trussell, 2018; McCormick, 1998), for instance via the exposure to maternally derived cortisol (Eriksen et al., 2006; Saino et al., 2005). For this reason, we also predict that (b) offspring born from predator‐exposed mothers will be smaller in size and will have shorter telomeres than offspring from control mothers.

## 2 MATERIALS AND METHODS

### 2.1 Fish population and experimental design

Individuals used in this study were descendants of wild‐caught guppies from a high predation site of the Tacarigua river in Trinidad (National Grid Reference: SP 787 804) in 2002. Since 2013, these fish are maintained as a self‐sustaining population kept under seminatural conditions in a 4,600 × 440 cm (40 cm filled with water) pool at the Botanical Garden of the University of Padova. We used a total of 160 females born in May 2017 and raised in the laboratory at standard conditions (see below) in single‐sex tanks. When fully sexually mature (6–7 months old), females were randomly assigned to 40 experimental tanks (50 × 27 cm and 15 cm filled with water), each housing four females. The bottom of the tanks was covered with mixed‐color gravel, while the walls of the tank were obscured with dark curtains, in order to minimize external disturbance to the fish. The tanks were subjected to a controlled photoperiod (12:12 hr light:dark cycle) and were maintained at 26 ± 1°C. All fish were fed ad libitum twice a day with a diet of fresh *Artemia salina* nauplii supplemented with commercial dry food. Half of the 40 tanks were assigned to the predation risk group and the other half to the control group. A supplementary tank with other virgin females was kept in the same room at the same conditions, allowing the replacement of dead fish to maintain the number of four fish per tank throughout the experiment. Females were allowed to acclimatize for 2 days. At the end of this period, a male was added to half of the female tanks (10 predation risk and 10 control tanks) and allowed to interact and mate with the females for 5 days. At the end of the mating period, males were allowed to rest for 2 days and subsequently added to the remaining half of the female tanks (10 predation risk and 10 control tanks), alternating the treatment groups. This allowed to control for paternal genotype between treatment groups (i.e., each of the 20 sires used in the experiment contributed equally to the offspring characteristics from the two treatment groups).

In order to manipulate the perception of the predation risk by the females, we used an experimental protocol previously used in this guppy population, which is known to elicit a strong antipredatory response (Evans, Gasparini, & Pilastro, 2007). In particular, we used four different models (size range 10.8–12.5 cm) that resembled the main natural predator of Trinidadian guppies, the cichlid *Crenicichla alta*, who prey predominantly on large and sexually mature size individuals (Magurran, 2005). We exposed female guppies to the predator model for 10 min three times per week, and we alternated each predator model among the predator tanks. The predator model was placed inside the tank and moved toward the end of the tank with jerky movements to increase its visibility as a threat (Dugatkin & Godin, 1992). In addition to the model predator, once a week we added 1 ml of conspecific alarm cue to each tank 2 min before inserting the predator in the tank. This conspecific alarm cue is generally released during a predator attack, because of the rupture of the prey epidermis, and was obtained following a similar protocol (Evans et al., 2007; Heathcote, Darden, Franks, Ramnarine, & Croft, 2017). Briefly, we obtained this cue by euthanizing 10 female guppies using an overdose of anesthetic (MS‐222); then, the tail, head, and internal organs were removed and the muscles were homogenized together with distilled water. The liquid was then filtered to avoid any particles and then centrifuged for 3 min.

We exposed females for 3 weeks; we stopped the exposure for 2 weeks; and then, we repeated the exposure for another 3 weeks. Thus, females perceived the risk of predation for a total of 6 weeks. Virgin females could be at different stages of ovarian cycle, that is, different stages of eggs maturation (Liley & Wishlow, 1974). A prolonged exposure to the stress ensured that the females perceived the risk of predation during the whole embryo development.

Throughout the experiment, on the day when the predator model was presented and the alarm cue was added to the treatment tanks, we recorded the behavior of the females in a subset of the experimental tanks. In particular, we focused on three typical antipredator behaviors that are elicited under predation risk: grouping behavior, freezing behavior, and predator inspection (Dugatkin & Godin, 1992; Heathcote et al., 2017; Seghers & Magurran, 1994). In accord with results from a previous experiment conducted in our laboratory (Evans et al., 2007), females exposed to the combination of predator model and alarm cues exhibited a strong antipredator response, by significantly increasing grouping and freezing behavior compared with controls and performing predator inspection throughout the duration of the experiment (Cattelan S., Panizzon P., Devigili A., Herbert‐Read J., Griggio M., Pilastro A., & Morosinotto C., unpublished data).

At the end of the treatment, all females were isolated and placed in a parturition tank with a separated nursery where newborns could easily escape from the mother to avoid cannibalism. Mothers produced a brood between 2 and 28 days after the end of the treatment (control: mean ± *SD* = 13.96 ± 7.96; predation risk: 13.09 ± 7.38; *t*_1,44_ = 0.384, *p* = .703).

We collected a maximum of three newborns per family (to account for the variance between siblings within family) when they were between 8 and 10 days old, we sacrificed them with an overdose of MS‐222 (ethyl 3‐aminobenzoate methanesulfonate salt) and stored in sterile microcentrifuge tubes at −80°C. Females were sacrificed after parturition (control: *N* = 35; predation risk: *N* = 24), if they did not give birth they were sacrificed within 70 days after the end of the treatment (control: *N* = 16; predation risk: *N* = 27). As for newborns, females were sacrificed with an overdose of MS‐222 and stored in sterile microcentrifuge tubes at −80°C.

### 2.2 Body size measurement

Each sacrificed fish was placed under a ZEISS Stemi 2000‐C stereomicroscope and photographed on its left side along with a scale for calibration, using a digital camera (Canon EOS 450D). Body size of each fish was calculated by measuring the distance between the snout and the base of the tail on the digital image using the ImageJ software (http://rsbweb.nih.gov/ij/download.html).

### 2.3 Relative telomere length measurement

Genomic DNA was extracted from the muscle of adult females and from the whole body of newborns. Extractions were performed using the EuroGOLD Tissue DNA Mini Kit (EuroClone S.p.A.) according to the manufacturer's protocol. Extracted DNAs were checked for yield and quality using a NanoDrop™ 2000c Spectrophotometer (Thermo Fisher Scientific Inc.) and by agarose gel electrophoresis, then stored at −20°C.

Relative telomere length (RTL) was measured using the real‐time qPCR method described by Cawthon (Cawthon, 2002), in which the relative telomere length is expressed, for each DNA sample, as the factor by which the sample differed from a reference DNA sample in its ratio (T/S) of telomere repeat copy number (T) to a single‐copy gene copy number (S). This ratio is proportional to the average telomere length. The telomere primers were the Tel1b (5′‐CGGTTTGTTTGGGTTTGGGTTTGGGTTTGGGTTTGGGTT‐3′) and Tel2b (5′‐GGCTTGCCTTACCCTTACCCTTACCCTTACCCTTACCCT‐3′) described in (Criscuolo et al., 2009). As a control single‐copy gene we used the melanocortin 1 receptor with the primers specific for guppies, MC1R‐F (5′‐GTCCTCGCTCTCCTTCCTGT‐3′) and MC1R‐R (5′‐CACACCACCGCGATGATGGT‐3′). We chose to use MC1R, because it is a single copy gene in many teleost species (Selz et al., 2007) and it has already been used in *Menidia menidia* for RTL calculation (Gao & Munch, 2015). Furthermore, the mapping of the primers on the guppy genome confirmed they would recognize a single region, corresponding to the MC1R gene. The glyceraldehyde‐3‐phosphate dehydrogenase (GAPDH), normally used as single‐copy gene in telomere studies, instead, seems to be duplicated in *P. reticulata* and indeed the GAPDH primers reported in the literature, recognized two regions in the guppy genome (A. Grapputo, personal observation). qPCRs were performed in triplicates in 96‐well plates, using an Applied Biosystems™ 7500 Real‐Time PCR System. Each well contained a total volume of 20 µl, including 4 µl of 5× HOT FIREPol^®^ EvaGreen^®^ qPCR Mix Plus (ROX) (Solis BioDyne), 5 ng of genomic DNA, and 200 nM of both forward and reverse primers. Telomere and MC1R amplifications were conducted on separate plates to maximize the number of samples per plate. qPCR profile consisted for both telomere and MC1R of one step at 95°C for 12 min followed by 40 cycles of 95°C for 20 s, 58°C for 18 s, and 72°C for 1 min. After each run was completed, a melt curve (15 s at 95°C, 1 min at 60°C, 15 s at 95°C, and 1 min at 60°C) was generated to confirm qPCR specificity. Each plate contained two interplate calibrators and a negative control, all run in triplicates. Baseline and cycle quantification (Cq) values were corrected and analyzed using the LinRegPCR software ver. 2017.1 (Ruijter et al., 2009). Cq correction across plates was done using a common threshold obtained from the reference DNA samples from all the plates (TEL threshold = 0.324; MC1R threshold = 0.346).

Relative telomere length was obtained following the equation proposed by Pfaffl (Pfaffl, 2001):

$$\text{RTL}=\frac{E_{\text{TEL}}^{\text{CqTEL}\left(\text{calibrator}\right)-\text{CqTEL}\left(\text{sample}\right)}}{E_{\text{MC}1R}^{\text{CqMC}1R\left(\text{calibrator}\right)-\text{CqMC}1R\left(\text{sample}\right)}}$$

where *E*_TEL_ is the mean efficiency of telomere plate; *E*_MC1R_ is the mean efficiency of MC1R plate; CqTEL(calibrator) and CqMC1R(calibrator) are the mean Cq values of the average of the two reference DNA samples in the plate, respectively, for telomere and MC1R; and CqTEL(sample) and CqMC1R(sample) are the mean Cq values for the triplicate of each sample in the plate, respectively, for telomere and MC1R. We set the acceptance threshold for amplifications efficiency to 100 ± 20%. Interassay coefficients of variation (CV) were 4.10% for telomere plates and 2.83% for MC1R plates, while intraassay CVs were 0.75% for telomere plates and 0.39% for MC1R plates.

### 2.4 Statistical analysis

Statistical analyses were performed using R v 3.5.2 (R Core Team, 2014). All variables were tested for normality and homogeneity of variance before analysis. First, we tested the effect of treatment on offspring body size (control: *N* = 67; predation risk: *N* = 58) by running a linear mixed‐effect model (LMM) with treatment fitted as fixed effect, the time between the end of treatment and birth and brood size as covariates, and mother and father identity as random factor. Father identity did not explain a significant part of the variance, and we thus dropped it from the model. We then tested the effect of predation risk on female RTL by running a LMM in which we fitted female RTL (control: *N* = 51; predation risk: *N* = 51) as dependent variable, treatment and parturition (0 or 1) as fixed effects, body size and the number of days between treatment and telomere measurement as covariates, and female tank as random effect. Female tank did not explain a significant part of the variance, and we thus dropped it from the model. We analyzed offspring RTL (control: *N* = 67; predation risk: *N* = 60) by performing a LMM fitting treatment as fixed effect, offspring body size, mother RTL and the number of days between treatment and birth as covariates, and mother and father identity as random effect. Father identity did not explain a significant part of the variance, and we thus dropped it from the model. LMMs were performed using the “lme4” R package (Bates, Mächler, Bolker, & Walker, 2015) fitted with Gaussian error distribution. The significance of fixed effects were calculated by means of chi‐squared tests using the “ANOVA” function in “car” R package (Fox & Weisberg, 2018) while the significance of random effects was tested using standard likelihood‐ratio tests with the “ranova” function in “lmerTest” R package (Kuznetsova, Brockhoff, & Christensen, 2017).

We tested for difference in RTL between females and offspring by running an independent *t* test using the mean offspring RTL of each family to balance the sample size between the two groups (females and offspring). We calculated the effect size (Hedges' *g*_s_) on the difference between treatments for offspring body size, female and offspring telomere length. SE of the effect size was calculated using a bootstrapping procedure based on 10,000 replications implemented in PopTools (Hood, 2010, version 3.2.5, available at http://www.poptools.org).

## 3 RESULTS

### 3.1 Offspring body size

**Figure 2:**
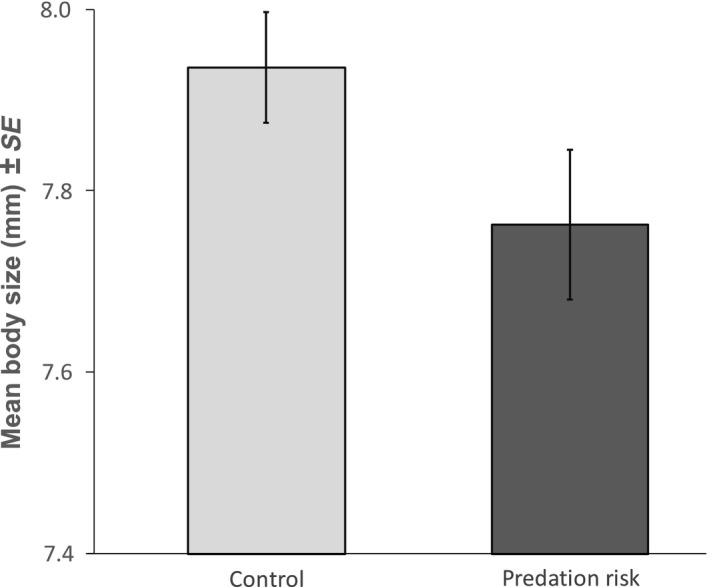
Body size of the offspring of the two experimental groups. Error bars represent the standard error of the mean observed values

Offspring produced by females that perceived the risk of predation were significantly smaller than their control counterparts (treatment: *χ* ^2^_1,42_ = 4.516, *p* = .034; Figure 2). Moreover, offspring body size was significantly influenced by the time between treatment and birth (*χ* ^2^_1,42_ = 19.102, *p* < .001) and, as expected, by brood size (*χ* ^2^_1,41_ = 11.967, *p* < .001). The effect of treatment on offspring body size, however, was not driven by differences between treatments neither in the brood size (*χ* ^2^_1,39_ = 2.132, *p* = .144) nor in the time between treatment and birth (*χ* ^2^_1,39_ = 3.054, *p* = .081). Finally, offspring size showed a significant amount of variance explained by mother identity (variance ± *SE* = 0.055 ± 0.011, *p* < .001).

### 3.2 Relative telomere length

**Figure 3:**
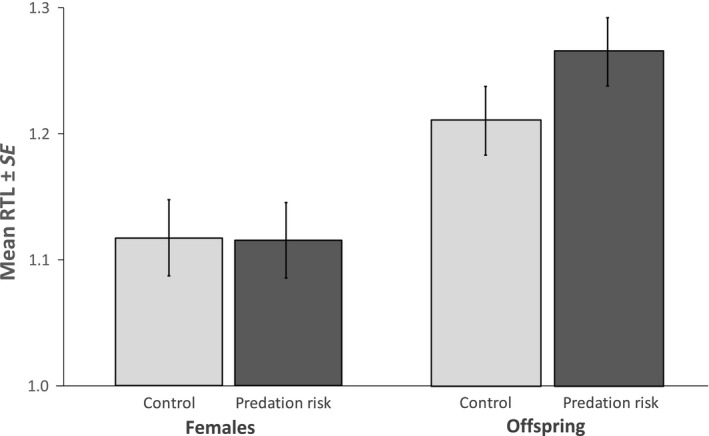
Relative telomere length of females and offspring of control and predation groups. Error bars represent the standard error of the mean observed values

Female RTL was not affected by the perceived risk of predation (*F*_1,91_ = 0.241, *p* = .625; Figure 3), parturition (*F*_1,91_ = 0.174, *p* = .678), body size (*F*_1,91_ = 0.471, *p* = .494), and the time between treatment and telomere measurement (*F*_1,91_ = 0.227, *p* = .635). Also, offspring RTL was not affected by maternal perceived predation risk (*χ* ^2^_1,35_ = 1.217, *p* = .270; Figure 3), maternal RTL (*χ* ^2^_1,34_ = 0.078, *p* = .780), the time between treatment and birth (*χ* ^2^_1,40_ = 0.715, *p* = .398), nor body size (*χ* ^2^_1,107_ = 3.684, *p* = .055). Offspring RTL showed a significant amount of variance explained by mother identity (variance ± *SE* = 0.038 ± 0.011, *p* < .001). Finally, females had significantly shorter RTL than offspring (*t*_1,84_ = −3.312, *p* = .001; Table 1; Figure 3), suggesting that RTL decreased with age, as found in many species.

**Table 1:** Mother and offspring RTL and offspring body size (standard length) of the two experimental groups

	Control		Predation risk		Hedges's *g*_s_ ±SE
	Mean ± *SD*	*N*	Mean ± *SD*	*N*	
Offspring body size	7.902 ± 0.377	67 (24)	7.772 ± 0.473	58 (22)	0.310 ± 0.187
Female RTL	1.117 ± 0.216	51	1.115 ± 0.214	51	0.001 ± 0.266
Offspring RTL	1.213 ± 0.252	67 (24)	1.269 ± 0.296	60 (22)	0.268 ± 0. 239

Note

The number of families is given in brackets. Effect sizes (Hedges' *g*_s_) with their standard errors of the difference between the two experimental groups are also given.

## 4 DISCUSSION

The prolonged predation risk perceived by females did not significantly affect their telomere length, nor that of their offspring, contrary to our predictions. We expected to observe shorter telomeres in females and their offspring exposed to predation risk, as several studies indicated that environmental stressors may enhance telomere attrition (reviewed in Chatelain et al., 2020). In our study, there was no effect of perceived risk of predation on neither females nor offspring telomere length (see also Kärkkäinen et al., 2019). There may be several explanations for this finding. One possibility is that the stress experienced by the mothers associated with our experimentally manipulated perception of predation risk was not sufficiently strong to determine a measurable telomere attrition. While this explanation cannot be ruled out, there are two reasons why it seems unlikely. First, females responded to predator models and to the alarm cue by showing the typical antipredator behavior observed in natural conditions and as found in previous laboratory experiments (Evans et al., 2007; Heathcote et al., 2017). Second, we found a significant reduction of offspring size at birth, which suggests that the stress experienced by the mothers during the gestation negatively impacted offspring as a reduced size constrains escape performance (Dial et al., 2016; Wolcott, Ojanguren, & Barbosa, 2017) and survival (Henrich, 1988) in fishes. Our result aligns with previous evidence in fishes in which the exposure to glucocorticoids (such as the cortisol) in the mother, and in turn in their eggs (Hwang, Wu, Lin, & Wu, 1992), was followed by the production of smaller offspring compared with offspring produced by undisturbed mothers (Eriksen et al., 2006; McCormick, 1998).

An alternative explanation may rest on the activity of telomerase, the enzyme that promotes the telomeric repair and reduces telomere erosion (Hatakeyama et al., 2008), which in fishes differs as compared with endotherm vertebrates. In endotherms, telomerase is generally suppressed in most of adult somatic tissues, an evolutionary response to the risk of tumor development because of endotherms’ higher metabolic rate and cellular replication (Olsson et al., 2018). By contrast, telomerase is expressed throughout the entire life in various teleost species (Hartmann et al., 2009; Hatakeyama et al., 2008; Lund et al., 2009). Thus, it is possible that a high telomerase activity masked the effect of stress on telomere attrition in our experiment. Furthermore, telomerase has been shown to be upregulated during regeneration of injured tissues in several ectotherm vertebrates (Anchelin, Murcia, Alcaraz‐Pérez, García‐Navarro, & Cayuela, 2011; Elmore et al., 2008). Laboratory studies on rats have also shown that telomerase activity is upregulated in response to chronic stress (Beery et al., 2012), suggesting a potential role of telomerase into resilience to stress, in accord to what has been reported for humans (Wolkowitz et al., 2012). Despite the prolonged activity of telomerase through fish life stages, telomerase compensatory activity is probably incomplete as many teleost species show a gradual telomere shortening over time similarly to what found in other vertebrates (Hartmann et al., 2009; Hatakeyama et al., 2008; Rollings, Miller, & Olsson, 2014). However, it is possible that a short‐term increase in telomerase activity masked the effect of our treatment on telomere length, because it was measured when the mothers produced a brood, which occurred between 2 and 28 days after the end of the treatment. It is therefore possible that predation risk had only a transient effect on telomere length. Clearly, investigations into the expression of telomerase in guppies, both during exposure to stress and during different life stages, are needed to test this hypothesis.

Although maternal telomere length was not related to offspring telomere length, we found a significant effect of mother's identity on offspring telomere length suggesting that the within‐brood variance in telomere length was lower than that among broods. Since paternal effects were balanced between groups, our finding suggests that maternal effects are a source of variation in offspring telomere length although it was unrelated to our experimentally induced predation risk perceived by the mothers. In the great reed warblers, *Acrocephalus arundinaceus*, it has also been found a strong maternal effect on offspring telomere length at birth (Asghar et al., 2015), but the possible underlying factors are unknown. Opposite to our results, in the Atlantic salmon (*Salmo salar*) a significant paternal but not maternal effect on offspring telomere length has been found (McLennan et al., 2016) confirming the extreme variation in telomere dynamics among teleosts (Olsson et al., 2018). Finally, we found that newborn guppies have longer telomeres than their mothers. This finding suggests a shortening of telomeres from birth to adulthood, in line with observations in other teleost species and with other vertebrates where telomere length gradually decreases with age (Haussmann et al., 2003; Ocalewicz, 2013).

Our study on guppies shows that the predation risk perceived by the mothers during gestation negatively affects offspring size at birth, but not their telomere length. At the same time, a significant maternal effect on offspring telomere length was also apparent. Future investigations of telomere dynamics are necessary to understand how environmental stress affects telomere length in fish, and whether telomere length is associated with senescence and life span in this group of vertebrates.

## CONFLICT OF INTEREST

The authors have no conflict of interests.

## AUTHOR CONTRIBUTIONS

AG and AP, with input from CM and SC, conceived the experimental design; SC and CM performed the predation experiment; SM did the laboratory work and performed the telomere analysis; SC performed the statistical analysis; SM and SC wrote the first draft of the manuscript; all the authors contributed critically to the writing of the manuscript.

## Data Availability

The dataset supporting this article is available at https://datadryad.org/review?doi=doi:10.5061/dryad.kt66rj0 (Monteforte, Cattelan, Morosinotto, Pilastro, & Grapputo, 2019).
